# Phenotype, Virulence and Immunogenicity of *Edwardsiella piscicida* Cyclic AMP Receptor Protein (Crp) Mutants in Catfish Host

**DOI:** 10.3390/microorganisms8040517

**Published:** 2020-04-04

**Authors:** Peng Zhou, Xueqing Han, Xiang Ye, Feifei Zheng, Ting Yan, Quan Xie, Yong-An Zhang, Roy Curtiss, Yang Zhou

**Affiliations:** 1 Department of Aquatic Animal Medicine, College of Fisheries, Huazhong Agricultural University, Wuhan 430070, China; 15926316870@163.com (P.Z.); xqhan94@163.com (X.H.); 18086042051@163.com (X.Y.); zhengfeifei@mail.hzau.edu.cn (F.Z.); 635412738@webmail.hzau.edu.cn (Q.X.); yonganzhang@mail.hzau.edu.cn (Y.-A.Z.); 2 College of Food Science and Technology, Huazhong Agricultural University, Wuhan 430070, China; yt15827390282@163.com; 3 Department of Infectious Disease and Immunology, College of Veterinary Medicine, University of Florida, Gainesville, FL 32608, USA

**Keywords:** *Edwardsiella piscicida*, cAMP receptor protein, virulence, vaccine

## Abstract

*Edwardsiella piscicida*, a facultative aerobic pathogen belonging to the Enterobacteriaceae family, is the etiological agent of edwardsiellosis that causes significant economic loses in the aquaculture industry. cAMP receptor protein (CRP) is one of the most important transcriptional regulators, which can regulate large quantities of operons in different bacteria. Here we characterize the *crp* gene and report the effect of a *crp* deletion in *E. piscicida*. The *crp*-deficient mutant lost the capacity to utilize maltose, and showed significantly reduced motility due to the lack of flagella synthesis. We further constructed a ΔP_crp_ mutant to support that the phenotype above was caused by the *crp* deletion. Evidence obtained in fish serum killing assay and competitive infection assay strongly indicated that the inactivation of *crp* impaired the ability of *E. piscicida* to evade host immune clearance. More importantly, the virulence of the *crp* mutant was attenuated in both zebrafish and channel catfish, with reductions in mortality rates. In the end, we found that *crp* mutant could confer immune protection against *E. piscicida* infection to zebrafish and channel catfish, indicating its potential as a live attenuated vaccine.

## 1. Introduction

*Edwardsiella piscicida* is a facultative anaerobe and Gram-negative enteric pathogen that generally causes lethal edwardsiellosis, which is a systematic enterohemorrhagic septicemic disease that can lead to high stock mortality in fresh and marine fish [1,2]. *E. piscicida* infection induces symptoms including emphysematous putrefactive disease with swelling skin lesions, as well as gill ulceration and necrosis in internal organs such as kidney, liver, spleen, and musculature [2,3].

To date, antimicrobial susceptibility testing shows that *E. piscicida* strains isolated from different hosts and geographical regions are susceptible to most commonly used antibiotics for the treatment of edwardsiellosis [4]. However antibiotic abuse represents a problematic method of treating bacterial infections in the aquaculture industry and has led to the evolution of multi-drug resistance strains [5]. Plasmid-mediated multi-drug resistant has been previously reported in *E. piscicida* [6]. In response to this potential, vaccination is an important disease control strategy that has significantly contributed to reduction of outbreaks and antibiotics use in aquaculture [7]. It is worth noting that intracellular bacterial vaccines evoke cellular mediated immune responses that “kill” and eliminate infected cells [7]. The intracellular lifestyle of *E. piscicida* has been gradually revealed. This pathogen could successfully survive and replicate within fish phagocytes [8]. In summary, the above attributes make *E. piscicida* a promising candidate to be developed as a live attenuated vaccine against edwardsiellosis.

The cyclic AMP receptor protein (Crp), also called catabolite gene activator protein (CAP), is member of the CRP-FNR (fumarate nitrate reductase regulator) superfamily of transcription factors [9]. In the lack of a preferred carbon source (e.g., glucose), the Crp-cAMP complex contributes to the phenomenon of catabolite repression, inducing catabolic pathways for growth on alternative substrates [10]. Crp enhances the ability of the RNA polymerase holoenzyme to bind and initiate the transcription of specific sets of genes. In *Escherichia coli*, there are currently estimated to be more than 100 operons and ~500 genes under the control of Crp-cAMP [11]. The Crp-cAMP complex also contributes to the regulation of virulence gene expression in many pathogenic bacteria including *Salmonella*, *Vibrio cholerae*, *Yersinia*, and *Mycobacterium tuberculosis* [12,13,14,15]. *crp* deletion mutants have been successfully developed as live attenuated bacterial vaccines to protect various animal species against different bacterial pathogens [12,16,17,18]. In fish pathogens, mutation of *crp* in *Aeromonas salmonicida* attenuated its virulence as ~6 times in *Oncorhynchus mykiss.* And the Δ*crp* mutant could confer protective immunity against the intraperitoneal challenge with *A. salmonicida* wild type [19]. And in *E. ictaluri*, *crp* mutant was also attenuated and conferred immune protection against *E. ictaluri* challenge to zebrafish (*Danio rerio*) and catfish (*Ictalurus punctatus*) [20]. In this study, we investigated the effects of a *crp* gene deletion on *E. piscicida* physiology, virulence and ability to confer immune protection to fish hosts.

## 2. Materials and Methods

### 2.1. Ethics Statement

All animal experimental work was approved by the Laboratory Animal Monitoring Committee of Huazhong Agricultural University. Procedures conducted involving in animals were in accordance with the suggestions of the Guide for the Care and Use of Laboratory Animals of Hubei Province, China.

### 2.2. Bacterial Strains and Growth Conditions

The bacterial strains used in the study are listed in Table 1. *E. piscicida* was routinely cultured in Luria broth (LB) or on Luria agar (LA) plates (Difco, Detroit, MI, USA) at 28 °C. When necessary, 0.2% arabinose was supplemented to activate the P_BAD_. *Escherichia coli* χ7213 was used for mutant plasmid harvest and was cultured in LB broth at 37 °C, supplemented with diaminopimelic acid (50 g/L) (Sigma, St. Louis, MO, USA). When necessary, ampicillin (Sigma, St. Louis, MO, USA) and chloramphenicol (Sigma, St. Louis, MO, USA) were supplemented at final concentrations of 100 and 50 μg/mL, respectively.

### 2.3. Sequence Analysis

The *crp* nucleic acid and Crp amino acid sequences from various bacteria species were downloaded from NCBI. The accession numbers were provided in the supplementary data. MAFFT 7 and ESPript 3 were used for multiple sequence alignment. The neighbor-joining method in Molecular Evolutionary Genetics Analysis package (MEGA 7.0) was used to construct a phylogenetic tree. The evolutionary distances were computed using the p-distance model. The *E. piscicida* Crp 3D structure was predicted using the Iterative Threading ASSEmbly Refinement (I-TASSER) server [21].

### 2.4. Construction and Characterization of Crp Mutants

Primers and plasmids used in this experiment are listed in Table 1 and Table 2. The procedures for mutant construction were described previously [22]. The Primers P1/P2 and P3/P4 were used in amplifications of upstream or downstream flanking fragments of *crp* via PCR respectively. Then, the upstream and downstream homologous arms were fused by overlap PCR using primers P1/P4, and ligated into the suicide plasmid pRE112 at the *Xba*I/*Sac*I sites. The resulting plasmid pRE112-*crp* was transformed into *E. coli* χ7213 for mobilization into *E. piscicida* via conjugation. Strains containing single-crossover plasmid insertions were isolated on LA media containing chloramphenicol. Loss of the suicide vector after the second recombination between homologous regions was selected by using the *sac*B-based sucrose-sensitivity counter-selection system. Resulting strain was selected on LA plates containing chloramphenicol. PCR identification via primers P1/P4 and direct DNA sequencing of the mutation sites using genomic DNA preparations were conducted to verify that the construction of the *crp* gene deletion strain was completed.

We further constructed ΔP_crp_ by replacing the promoter of *crp* gene with the arabinose-regulated *ara*C P_BAD_ promoter. The procedures were described previously [23]. A 557-bp DNA fragment containing the region upstream of the *crp* promoter was PCR amplified as a template with primers P5/P6 (Table 2). The PCR-amplified fragment was digested with *Bgl*II/*Hind*III and cloned into the vector pYA3700 (Table 1). A 595-bp PCR fragment, including 583-bp of *crp* gene and its original Shine-Dalgarno sequence was amplified using primers P7/P8 (Table 2). The PCR fragment was digested with *Xho*I/*Kpn*I and inserted into the intermediate plasmid described above. The resulting construct was confirmed by DNA sequence analysis. Then, a 2.5-kb DNA fragment including *ara*C P_BAD_, P_crp_ 5’ and 3’ flanking regions were excised from the plasmid by digestion with *Kpn*I/*Xma*I and inserted into pRE112, resulting in plasmid pRE112-ΔP_crp_. The following procedure was the same as that in Δ*crp* mutant construction.

To determine the growth kinetics of different strains, 1:100 diluted overnight cultures were cultured in LB medium at 28 °C. Samples were taken hourly, and the optical densities were measured at 600 _nm_ (OD_600__nm_). In addition, on 2 h, 4 h, 6 h, 8 h, 10 h, 12 h, 24 h, 36 h post inoculation, the numbers of CFU in cultures were determined by serial dilutions and plating.

In order to study whether CRP is involved in the process of maltose utilization, the growth of *crp* mutant strains and parent strain were observed on MacConkey agar supplemented with maltose (1%). Swimming motility was measured on LA plates that were prepared with 0.3% (*w/v*) agar according to the method described previously [20]. To increase flagella synthesis, the bacterial samples were collected from motility agar plates. Negative staining method was used to observe the bacteria morphology under transmission electron microscopy (TEM; Hitachi H-7000FA, Tokyo, Japan). Ten microliters of the resuspension of each strain was applied to Formvar-coated copper grids and negatively stained for 1 min with 1% uranyl acetate [20].

### 2.5. Resistance Against Host Clearance of E. piscicida

To investigate resistance of the parent J118 strain and Δ*crp* against serum killing, a catfish serum survival assays was accomplished as previously described [24]. Blood was withdrawn from the caudal vein of channel catfish (average weight, 500 g) by sterile injector and placed on ice immediately. The blood was allowed for clotting over night at 4 °C. For serum survival assay, the serum was treated with or without heating at 56 °C for 1h. A mid-log growth phase inoculum of 5.0 × 10^5^ CFU bacteria in 100 μL was mixed with 400 μL serum. The mixtures were incubated at 28 °C for 1h. Then, the numbers of CFU were determined by serial dilutions and plating. The survival percentage was subsequently calculated as follows: (CFUs after co-incubation /CFUs in PBS control group) × 100%. *E. coli* DH5α strain was used as a sensitive control strain in this assay. The experiments were repeated three times independently.

To determine the persistent carrier state of J118 and Δ*crp* strains in host, the competitive assay was performed as previously described [22]. J118 and Δ*crp* strains grown to mid-exponential phase in LB were harvested by centrifugation, washed and resuspended in PBS to a bacterial density of 5.0 × 10^6^ CFU/mL, respectively. In three independent assays, a total of eleven channel catfish were injected by the intracoelomic (i.c.) route with 200 μL volume of 1:1 Δ*crp* mutant – J118 mixture (1.0 × 10^6^ CFU/fish). Twenty-four hours later, fish were euthanized and the blood, ascites, liver, spleen, trunk kidney and head kidney samples were collected. Homogenates were serially diluted and plated on MacConkey agar with maltose (1%) at 37 °C for 24 h. The parent strain is red and the Δ*crp* mutant is white on the plate, respectively. Bacterial counts titers were calculated by dividing the weights of the tissues from the bacteria loads in the samples.

### 2.6. Virulence of E. piscicida Δcrp in Zebrafish and Catfish Fingerlings

The zebrafish (average weight, 300 mg) used in this study were from the Institute of Hydrobiology, Chinese Academy of Sciences (Wuhan, China). The zebrafish were acclimatized for 2 weeks before injection. They were fed with commercial feed for aquatic animal twice per day under natural photoperiod. The water temperature was maintained at 24–26 °C during cultivation. Zebrafish are divided into 12 groups, each group of 10, infected with J118 strain and Δ*crp* respectively (doses: 10^2^ CFU/fish, 10^3^ CFU/fish, 10^4^ CFU/fish, 10^5^ CFU/fish, 10^6^ CFU/fish, and 10^7^ CFU/fish). Chanel catfish (average weight, 800 mg) from a local aquaculture farm were acclimatized and monitored for 2 weeks before infection. Catfish were fed daily with commercially produced food pellets (Haida, China) under natural photoperiod. Water temperature was maintained at 23–25 °C. Catfish are divided into 10 groups, each group of 10, i.c. infected with J118 strain and the Δ*crp* mutant, respectively (doses: 10^4^ CFU/fish, 10^5^ CFU/fish, 10^6^ CFU/fish, 10^7^ CFU/fish, and 10^8^ CFU/fish). The clinical symptoms and mortality of infected fish were observed for 14 days. The LD_50_ was calculated by Karber’s methods [25].

### 2.7. Immune Protection of E. piscicida Δcrp Mutant in Zebrafish and Catfish Fingerlings

We further evaluated the immune protection mediated by Δ*crp* in zebrafish and catfish fingerlings. Fish were divided into immunized group and control group, with 10 zebrafish in each group and 20 catfish fingerling in each group. Zebrafish and catfish were i.c. immunized with 10^3^ CFU/fish and 10^4^ CFU/fish of Δ*crp*, respectively. Subsequent booster immunization was given at day 14 post-primary immunization. At 2 weeks post-booster immunization, zebrafish and catfish were challenged i.c. with 10-times LD_50_ *E. piscicida* J118. The fish were observed for clinical symptoms and mortalities for 14 days.

### 2.8. Statistical analysis

Statistical analysis was performed by GraphPad Prism 6 (Graph Pad Software, Inc., Graph Pad Software, Inc., San Diego, CA, USA). Survival data were analyzed with the log-rank (Mantel-Cox) test. The statistical p values were calculated by the two-tailed Mann-Whitney t test. Differences were considered significant at *p* < 0.05 and highly significant at *p* < 0.01.

## 3. Results

### 3.1. Sequence Analysis

**Figure 1:**
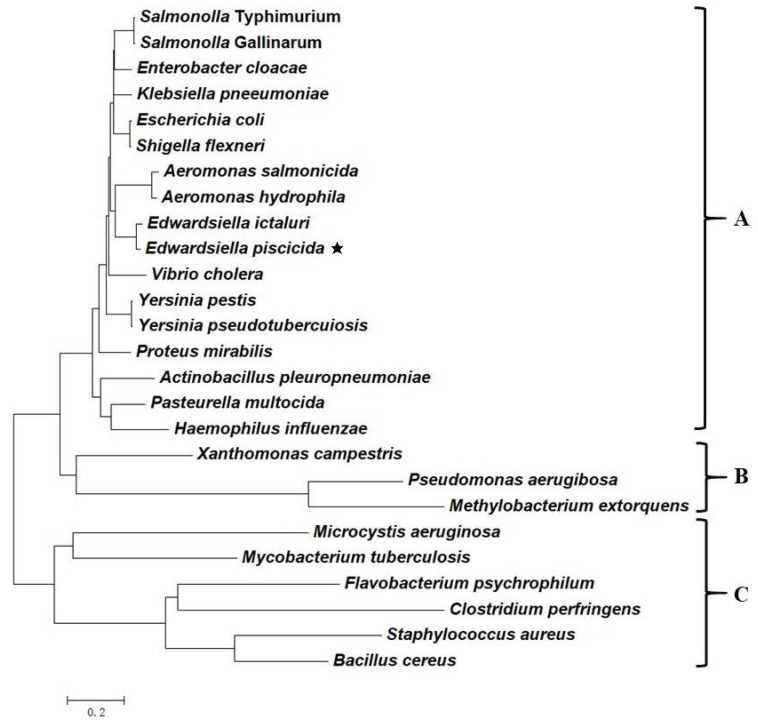
Phylogenetic tree of *crp* gene. The evolutionary history was inferred using Molecular Evolutionary Genetics Analysis (MEGA) 7.0 with neighbor-joining method. The tree is drawn to scale, with branch lengths in the same units as those of the evolutionary distances used to infer the phylogenetic tree. The analysis involved 26 nucleotide sequences. *E. piscicida crp* was marked with ★.

The catabolic and virulence regulator *crp* gene is wide spread not only between enteric pathogens, including *E. piscicida*, but also within other bacterial domains (Figure 1). The phylogenetic tree clearly showed *crp* genes in Enterobacteriaceae, Aeromonadaceae, Vibrionaceae and Pasteurellaceae, belonging to γ-Proteobacteria, were clustered in clade A. The closely related clade B includes other Proteobacteria, while the clade C includes Cyanobacteria, Actinobacteria, Bacteroidetes and Firmicutes, with a more distantly evolutionary relationship. Especially, *crp* genes in enteric fish pathogens including two *Aeromonas* species and two *Edwardsiella* species showed close genetic relationship according to this phylogenetic tree.

**Figure 2:**
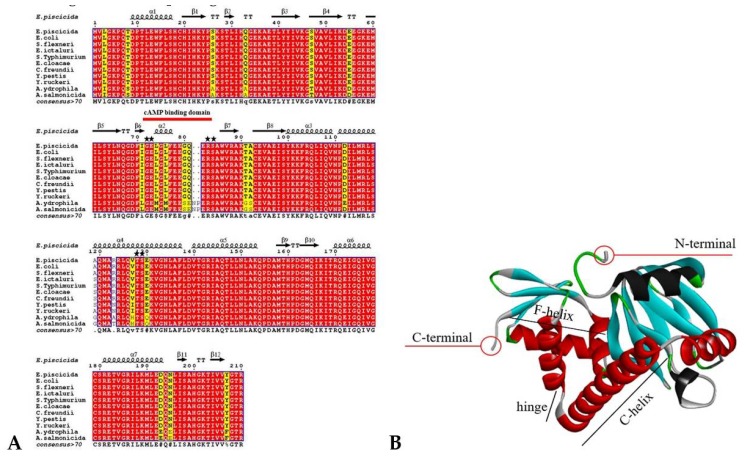
Multiple alignments of Crp and 3-D predicted structure. (**A**) Secondary structure of *E. piscicida* Crp and alignment between representative Crp proteins. The secondary structure at the top of the alignment corresponds to the *E. piscicida* Crp (spirals represent α-helix; arrows represent β-sheet). Conserved amino acids residues are indicated in red. The stars indicate the amino acid residues required for cAMP binding. (**B**) *E. piscicida* Crp monomer structure. The helixes required for DNA binding and dimer Crp formation are indicated (C-helix, F-helix, and hinge). The N- and C-termini are indicated in a red circle.

Sequence and structural alignment between functional representative bacterial Crp proteins revealed that 189 amino acid residues (90%) are strictly conserved out of 210 residues in *E. piscicida* Crp (Figure 2A). *E. piscicida* Crp has 100.0%, 99.0%, 98.6%, and 89.0% amino acid similarity to the Crp of *Escherichia coli*, *E. ictaluri*, *Yersinia pestis* and *Aeromonas hydrophila*, respectively. The conserved cAMP binding amino acid residues: Gly72, Glu73, Arg83, Ser84, Thr128, and Ser129 (Figure 2A) of *E. piscicida* Crp is similar to other Gram-negative Crp-family members. The *E. piscicida* Crp 3-D predicted structure exhibits the flexible hinge required for Crp dimerization and the F-helix & C-helix that interacts with the DNA (Figure 2B). Thus, above results shows that Crp is conserved and indicates its function is conserved through the evolution of the Enterobacteriaceae family, including enteric fish pathogens.

### 3.2. Construction and Characterization of Crp Mutants

**Figure 3:**
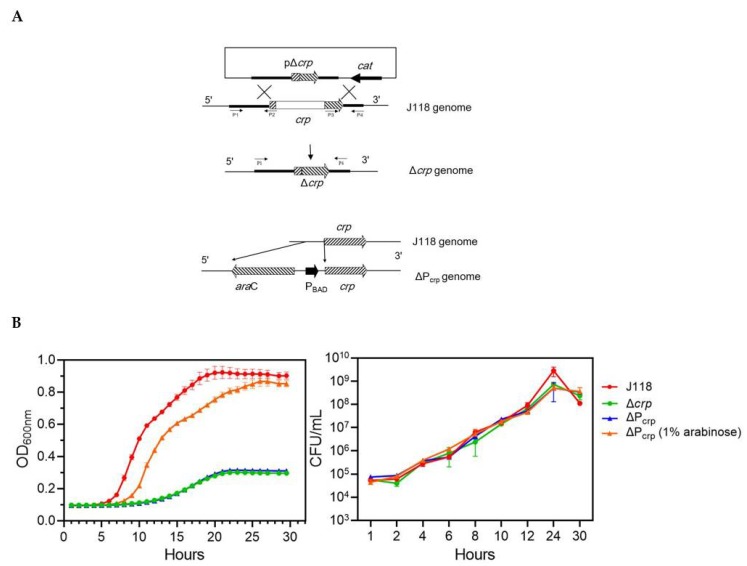
Construction and growth curves of *crp* mutant*s*. (**A**) Strategy for construction of Δ*crp* and ΔP_crp_ by homologous recombination. (**B**) Growth curves in LB medium over a 30 h period. Data are presented as the averages ± the standard deviations for three replicates.

**Figure 4:**
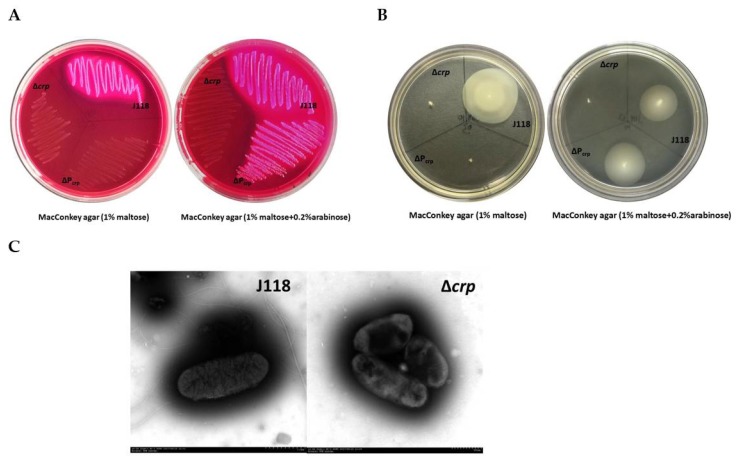
Phenotype characterization of *crp* mutant*s*. (**A**) Phenotype verification on MacConkey agar plates supplemented with 1% maltose and with or without 0.2% arabinose. (**B**) Swimming zones through 0.3% LB agar with or without 0.2% arabinose. (**C**) Negative staining-transmission electron microscopy of *E. piscicida* parent strain J118 (scale bar = 1 μm) and *crp* mutant (scale bar = 500 nm).

To investigate the role of Crp in the pathogenesis of *E. piscicida*, a *crp* deletion mutant was constructed by allelic replacement in parent strain J118 (Figure 3A). The growth of *E. piscicida* strains was quantified over time by measuring the OD_600__nm_ and CFUs in cultures (Figure 3B). Although the OD_600__nm_ growth curve showed growth defect of Δ*crp* in LB broth, the CFUs showed no differences between Δ*crp* mutant strain and parent strain, which indicated that the mutation of *crp* affected the secretion of bacterial extracellular products. The phenotype by growth on MacConkey agar supplemented with maltose (1%) shows that the Δ*crp* mutant does not utilize maltose, indicating that the genes related to maltose utilization is under the positive regulation by Crp (Figure 4A). Except for involvement in catabolic functions, the Crp regulatory complex is also necessary for the flagella synthesis. We evaluated the motility of the Δ*crp* mutant in LB broth supplemented with 0.3% agar and flagella synthesis by TEM. The results showed that the Δ*crp* mutant loses motility (Figure 4B) due to the lack of flagella synthesis (Figure 4C), indicating that Crp positively regulates flagella synthesis in *E. piscicida.*

We further constructed a ΔP_crp_ mutant by replacing the promoter of *crp* gene with the arabinose-regulated *ara*C P_BAD_ promoter (Figure 3A). The transcription of *crp* is dependent upon arabinose availability. The ΔP_crp_ showed similar phenotypes with Δ*crp* mutant in the absence of arabinose (Figure 3B, Figure 4A,B). However, when supplemented with arabinose, the defects in growth, maltose utilization and motility were all well complemented (Figure 3B, Figure 4A,B). Above results verify that *crp* mutation, instead of other random mutation, contributes to the phenotypes differences.

### 3.3. Resistance Against Host Clearance of E. piscicida Δcrp

**Figure 5:**
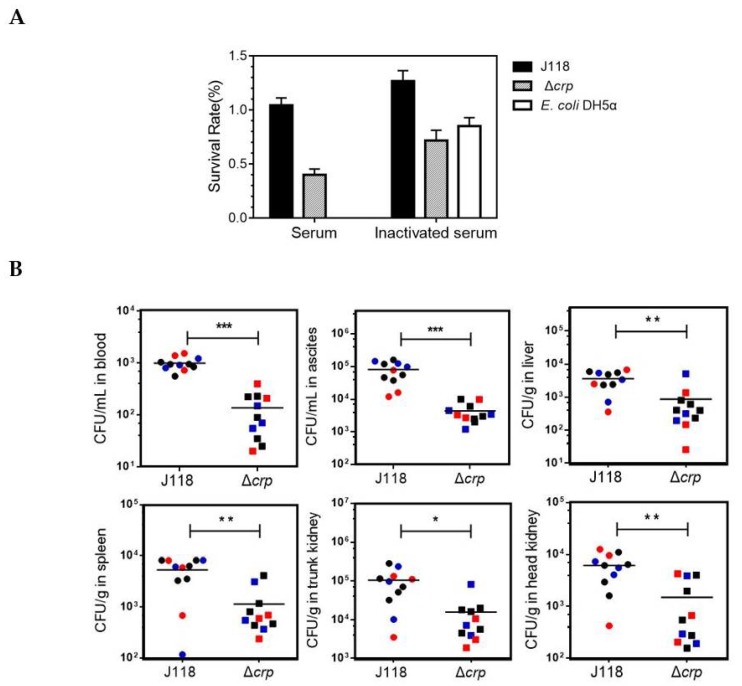
Effect of Crp deletion on resistance against host clearance. (**A**) Percentage of CFUs following 1 h of incubation with catfish serum, or heat-inactivated catfish serum. (**B**) Colonization of channel catfish tissues by *E. piscicida* J118 and Δ*crp* mutant in competitive infection assay. The data was a combination the three independent assays. * *p* < 0.05, ** *p* < 0.01, *** *p* < 0.001.

To investigate the role of *crp* in *E. piscicida* resistance against innate immune responses, we first examined the abilities of the strains to survive in a channel catfish serum survival assay. When *E. piscicida* J118 strain was incubated with catfish serum, the survival rate of the bacteria was found to be 104.3%, revealing that parent strain resisted the bactericidal effect of catfish serum. Δ*crp* mutant had showed a significantly reduced survival rate (40.0%) compared with the parent strain (Figure 5A). The survival rate of *E. coli* DH5α, a serum-sensitive laboratory strain incubated under the same condition, was 0%. When incubated with inactivated serum, the Δ*crp* mutant showed an increased survival rate of 71.6%, but still lower than parent strain. Above results suggested that the *crp* mutant could poorly resist the killing by bactericidal substances in fish serum, and then could not effectively evade the host clearance.

To test this idea, we performed a competitive infection assay. An approximately 1:1 mixture of J118 and Δ*crp* bacteria was inoculated by the intracoelomic (i.c.) route into channel catfish. Twenty-four hours later, blood, ascites, liver, spleen, trunk kidney and head kidney samples were collected after the fish were euthanized. Comparing to the parent strain, Δ*crp* colonized at significantly lower levels in all the samples (Figure 5B), which confirmed that the *crp* mutation impaired the resistance ability of *E. piscicida* against innate immune clearance in vivo.

### 3.4. Virulence of E. piscicida Δcrp in Zebrafish and Catfish Fingerling

**Figure 6:**
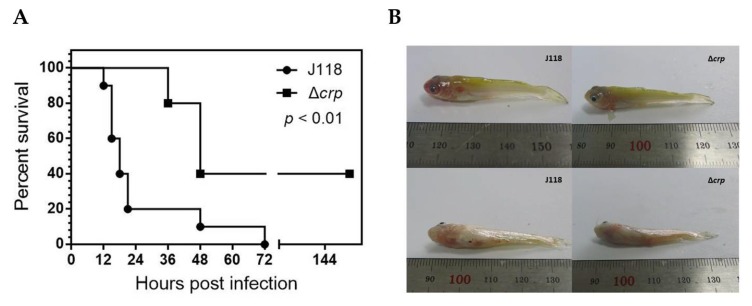
(**A**) Survival curves for zebrafish challenged with *E. piscicida* J118 and Δ*crp* mutant (1.0 × 10^6^ CFU). (**B**) Catfish fingerlings i.c. infected with *E. piscicida* J118 and Δ*crp* mutant (1.0 × 10^6^ CFU).

The ideal live attenuated bacterial vaccine should be totally attenuated. Zebrafish have been developed as an easy and powerful model to test pathogenesis of *E. piscicida.* The LD_50_ of the parent strain J118 was 7.94 × 10^3^ CFU, while the LD_50_ of the Δ*crp* mutant increased up to 3.16 × 10^5^ CFU, nearly 40-fold compared to the parental strain (Table 3). For the zebrafish infected with J118, the death occurred immediately during the first 24 h, while it was quite delayed in the Δ*crp* infection group (Figure 6A). The *E. piscicida* Δ*crp* mutant was further evaluated in the channel catfish host. We found that the Δ*crp* mutant applied by the i.c. route was attenuated with an estimated 10-times LD_50_ increase over the parent strain (Table 4). Catfish fingerlings that were infected with J118 developed the typical symptoms of enteric septicemia, skin lesions and distended abdomen, while the ones infected with the Δ*crp* mutant exhibited mild symptoms (Figure 6B). Taken together, these results indicate that Crp significantly contributed to the virulence of *E. piscicida*.

### 3.5. Immune Protection of E. piscicida Δcrp Mutant in Zebrafish and Catfish Fingerling

**Figure 7:**
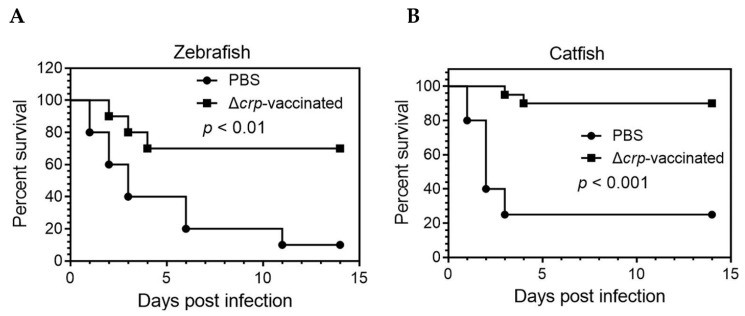
Immunization with Δ*crp* protects zebrafish (**A**) and channel catfish (**B**) against lethal challenges with parent strain. The data shown are representative of the results of one of three independent experiments; the survival data were analyzed using the log rank (Mantel-Cox) test.

Previous results indicate that the *E. piscicida* Δ*crp* mutant had reduced virulence and that the host is able to control the infection. We further evaluated the immune protection mediated by the Δ*crp* mutant in zebrafish and catfish fingerlings. At 2 weeks post-booster immunization, zebrafish were challenged i.c. with 10-times LD_50_ *E. piscicida* J118. The Δ*crp* mutant applied by the i.c. route was immune protective in the zebrafish host, with 70% *survival* while only 10% survival in the PBS control group (Figure 7A). A challenge study was also performed to determine whether the Δ*crp* mutant induces protective immunity in catfish fingerlings. At 2 weeks post-booster immunization, catfish fingerlings were challenged i.c. with 2-times LD_50_ *E. piscicida* J118. The Δ*crp* mutant showed a 90% survival rate against J118 infection, which is significantly higher than that of PBS (Figure 7B).

## 4. Discussion

cAMP receptor protein (CRP; also known as catabolite activator protein, CAP) is a regulatory protein in bacteria. The binding with cAMP causes a conformation change that allows CRP to bind tightly to the DNA site in the promoters of the genes it controls and interact with RNA polymerase [26,27]. Crp is widely distributed among the bacteria and relatively conserved, indicating its regulation of similar gene families related to carbohydrate metabolism [10,28], competence, growth, and virulence determinants [20,29,30,31].

*E. piscicida* is a facultative aerobic pathogen belonging to the Enterobacteriaceae family, with a wide ecological niche and host range including various species of fish [1]. In this study, a *crp* gene deletion mutant (Δ*crp*) and a *crp* promoter mutant (ΔP_crp_) of *E. piscicida* were constructed in order to investigate the role of the *crp* gene in *E. piscicida* physiology, virulence and ability to confer immune protection to fish hosts. Replacement of the promoter of *crp* gene by the arabinose-regulated *ara*C P_BAD_ promoter, yielding ΔP_crp_ in which *crp* transcription was arabinose dependent. In this study, ΔP_crp_ was used as complement strain when the arabinose was added. Firstly, the Δ*crp* mutant exhibited impaired growth *in vitro.* The growth delay in *crp* mutants seem common in diverse bacteria species, relating to its role as a global regulator [9]. Secondly, lacking of Crp, *E. piscicida* could not utilize maltose, which means that Crp does regulate the genes related to carbohydrate utilization and influence metabolism(s) in *E. piscicida*. In *E. coli*, the cAMP-CRP complex is required for *mal*T expression, the transcriptional activator of five transcriptional units related to the maltose regulon [32]. The regulation mechanism between Crp and maltose operon in *E. piscicida* still needs further investigation. Thirdly, the Δ*crp* mutant also loses motility which is conferred by flagella of bacterial pathogens [33]. Motility is critical for cell invasion and is associated with chemotaxis and secretion of effector proteins that act as virulence factors [34,35].

Considering this, we conducted a competitive infection assay and a fish serum suvival assay to compare systemic infection capacity of Δ*crp* and the parent strains. The Δ*crp* mutant showed poor colonization in different tissues and a weakened survival rate against catfish serum, indicating its attenuated evasion capacity against host immune clearance. In further virulence assessment assays, the LD_50_s of the Δ*crp* mutant in zebrafish and channel catfish increased by 40-fold and 10-fold, respectively, comparing to the parent strain, and Δ*crp* mutant infection group exhibited a delayed death (in the zebrafish) and mild symptoms (in the channel catfish). This was consistent with previous study that *E. piscicida* CK216, a Δ*crp* mutant strain exhibits 1000-fold attenuation comparing to wild type strain in gold fish [36]. Indeed, recent unpublished work in our laboratory indicates that Δ*crp* mutant could evoke host controllable inflammation which could effectively contribute to bacterial clearance, while J118 strain infection evoked excessive inflammatory cytokine production in the susceptible organ liver and trunk kidney, leading to severe tissue injury. Therefore, the above results support that Crp contributes to the virulence of *E. piscicida* and that the Δ*crp* mutant is attenuated.

Being a mucosal facultative intracellular pathogen, *E. piscicida* is an ideal candidate to develop a live attenuated vaccine for the aquaculture industry, which could elicit both humoral and cellular immune responses [37]. We further evaluated the immune protection mediated by the Δ*crp* mutant in zebrafish and catfish fingerlings. And as expected, the Δ*crp* mutant group had a significantly higher survival rate than the PBS control group. We determined that the Δ*crp* mutant could elicit host immune responses and offer moderate protection in zebrafish and catfish. Thence, the introduction of a Δ*crp* mutation could be considered as part of genetic plan to construct an *E. piscicida* live attenuated vaccine in further studies.

## 5. Conclusions

In summary, we conclude that Crp is involved in the regulation of maltose utilization and is required for flagella synthesis in *E. piscicida*. More importantly, Crp is also required for *E. piscicida* resistance against host immune elimination and that it contributes to the systemic dissemination and virulence of this bacterium. Finally, the Δ*crp* mutant could be a promising vaccine candidate for further genetic modification to control the *E. piscicida* infections.

## Figures and Tables

**Table 1:** Strains and plasmids used in this study.

	Description	Source or reference
**Strains** *Edwardsiella piscicida* EIB202 J118 Δ*crp* ΔP_crp_ *Escherichia coli*	CCTCC M208068, Col^r^, Cm^r^, pla^+^ pEIB202 curing derivative of EIB202, Col^r^, Cm^s^, pla^−^ *crp* deletion mutant ΔP_crp_TT *ara*C P_BAD_ *crp* TT	[6] Lab stocking J118 J118
χ7213	*thr*-1 *leu*B6 *fhu*A21 *lac*Y1 *gln*V44 *rec*A1 Δ*asd*A4 Δ(*zhf*-2::Tn10) *thi*-1	[16]
**Plasmids** pYA3700 pRE112	TT *ara*C P_BAD_ cassette plasmid; Ap^r^ Suicide vector, *sac*B, mob^−^(RP4)R6K ori, Cm^r^	[23]
pRE112-*crp* pRE112-ΔP_crp_	pRE112 derivative, designed for knockout of *crp*, Cm^r^ pRE112 derivative, designed for replacement of *crp* promoter, Cm^r^	pRE112 pRE112

Col^r^ stands for Colistin-resistant. Cm^r^ stands for Chloramphenicol-resistant. Ap^r^ stands for Ampicillin-resistant.

**Table 2:** Primers used in this study.

Primer	Sequence (5’-3’)	Product Size (bp)	Tm	Target Gene	Source or Reference
P1	CGCTCTAGACCACAGGACAAACCAAAACC	593	58	Upstream fragment for Δ*crp*	This study
P2	TGCTGGAGGATCAGAACCTGATCTCGGCACACGGTAAAAC			construction	
P3	CAGGTTCTGATCCTCCAGCAGTTGGATCTGTTTGCGGTTT	579	58	Downstream fragment for Δ*crp*	This study
P4	CCCGAGCTCAGAGACGCTGGATAGCCTGA			construction	
P5	CCCAGATCTTCTATACCCGCTTCATTCCA	557	58	Upstream fragment for ΔP_crp_	This study
P6	CGCAAGCTTCCCCGGGCCGTCCAATATCGAATACCA			construction	
P7	CGCCTCGAGGGATAATAGCGAATGGTTCTC	595	58	Downstream fragment for ΔP_crp_	This study
P8	CGCCTCGAGGGATAATAGCGAATGGTTCTC			construction	

**Table 3:** Calculations of LD_50_s of J118 and Δ*crp* mutant strains in zebrafish.

Dose of Challenge CFU	Number of Death/Total		Survival Rate (%)	
	J118	Δ*crp*	J118	Δ*crp*
1.0 × 10^7^	-	10/10	-	0
1.0 × 10^6^	10/10	6/10	0	40
1.0 × 10^5^	8/10	3/10	20	70
1.0 × 10^4^	6/10	1/10	40	90
1.0 × 10^3^	2/10	0/10	80	100
1.0 × 10^2^	0/10	-	100	-
LD_50_*	7.94 × 10^3^	3.16 × 10^5^		

* The LD_50_ was calculated according to Karber’s method.

**Table 4:** Calculations of LD_50_s of J118 and Δ*crp* mutant strains in channel catfish.

Dose of Challenge CFU	Number of Death/Total		Survival Rate (%)	
	J118	Δ*crp*	J118	Δ*crp*
1.0 × 10^8^	-	10/10	-	0
1.0 × 10^7^	10/10	7/10	0	30
1.0 × 10^6^	5/10	0/10	50	100
1.0 × 10^5^	2/10	-	80	-
1.0 × 10^4^	0/10	-	100	-
LD_50_*	6.31 × 10^5^	6.31 × 10^6^		

* The LD_50_ was calculated according to Karber’s method.

## Supplementary Materials

The NCBI accession numbers of Crps were as follows: *Edwardsiella piscicida* (WP_000242755.1); *Edwardsiella ictaluri* (WP_000242758.1); *Escherichia coli* (NP_417816.1); *Enterobacter cloacae* (WP_000242758.1); *Shigella flexneri* (NP_709132.1); *Salmonolla* Typhimurium (NP_462369.1); *Salmonolla* Gallinarum (WP_000242751.1); *Citrobacter freundii* (WP_000242758.1); Yersinia pestis (YP_002345259.1); *Yersinia ruckeri* (WP_004718520.1);*Yersinia pseudotubercuiosis* (WP_002212297.1); *Aeromonas hydrophila* (YP_855526.1); *Aeromonas salmonicida* (WP_005311762.1); *Klebsiella pneeumoniae* (WP_000242758.1); *Shigella flexneri* (NP_709132.1); *Vibrio cholera* (WP_000242749.1); *Proteus mirabilis* (WP_004246872.1); *Actinobacillus pleuropneumoniae* (WP_005618610.1); *Pasteurella multocida* (WP_005723550.1); *Haemophilus influenza* (NP_439118.1); *Pseudomonas aerugibosa* (YP_006960515.1); *Xanthomonas campestris* (NP_635866.1); *Flavobacterium psychrophilum* (YP_001295622.1); *Microcystis aeruginosa* (WP_002793798.1); *Clostridium perfringens* (WP_003455357.1); *Staphylococcus aureus* (WP_000138218.1); *Bacillus cereus* (NP_830249.1); *Mycobacterium tuberculosis* (NP_218193.1); *Methylobacterium extorquens* (WP_012255086.1).
